# Influence of Accelerated Solvent Extraction Conditions on the LC-ESI-MS/MS Polyphenolic Profile, Triterpenoid Content, and Antioxidant and Anti-lipoxygenase Activity of *Rhododendron luteum* Sweet Leaves

**DOI:** 10.3390/antiox9090822

**Published:** 2020-09-03

**Authors:** Marta Olech, Lena Łyko, Renata Nowak

**Affiliations:** Department of Pharmaceutical Botany, Medical University, 1 Chodźki Street, 20-093 Lublin, Poland; lenakopacz@onet.pl (L.Ł.); renatanowak@umlub.pl (R.N.)

**Keywords:** honeysuckle azalea, yellow azalea, Ericaceae, anti-inflammatory activity, antioxidants, flavonoids, phenolic acids, triterpenes, LC-MS, ASE

## Abstract

Evaluation of native plant resources and their efficient use is one of the current trends in phytochemistry. The main aim of the present study was to investigate the biological activities of different *Rhododendron luteum* Sweet leaf extracts obtained with the use of accelerated solvent extraction using different solvents and extraction temperatures. All extracts were subjected to bioactivity assays, which revealed considerable anti-lipoxygenase (23.07–90.13% lipoxygenase inhibition) and antiradical potential. All samples exhibited high 2,2-diphenyl-1-picrylhydrazyl (DPPH^•^) (234.18–621.90 mg Trolox equivalents (TE)/g) and 2,2′-azino-bis-3(ethylbenzthiazoline-6-sulphonic acid) (ABTS^•+^) (88.79–349.41 mg TE/g) scavenging activity, high antioxidant potential in the Oxygen Radical Absorbance Capacity (ORAC) assay (495.77–1011.59 mg TE/g), and moderate ion chelating (Fe2+) capacity. The chemical profile of each sample was determined using liquid chromatography/electrospray ionization triple quadrupole mass spectrometry (LC-ESI-MS/MS) and spectrophotometric procedures. Twenty-three compounds representing seven polyphenol subclasses were detected and quantified, including some phenolic acids and flavonoids that had not been previously reported for this plant material. It was shown that 5-*O*-caffeoylquinic acid, protocatechuic acid, catechin, quercetin and its glycosides (hyperoside, isoquercetin, quercitrin), and pentacyclic triterpenes were the dominant secondary metabolites in *R. luteum* leaves. The antioxidant activity was found to be strongly related to different polyphenol groups and total triterpene content, while the anti-lipoxygenase potential was highly dependent on catechin.

## 1. Introduction

Chronic inflammation and oxidative stress contribute to the emergence and development of many diseases, for example, cancer, cognitive dysfunctions, rheumatoid arthritis, or heart diseases. Plant secondary metabolites may exhibit direct and indirect anti-inflammatory and antioxidant potential, without showing severe adverse effects [1,2]. In contrast, many popular anti-inflammatory drugs may cause nagging or painful side effects, e.g., damage to gastric mucosa, reduced production of gastric acid, diarrhea, or nausea [3]. Therefore, searching for new natural and safe agents seems to be a promising alternative.

Many studies suggest that polyphenols, including flavonoids, possess significant antioxidant and anti-inflammatory potential. They can present many mechanisms of action, such as inhibition of the production of inflammation mediators: nitric oxide radicals, tumor necrosis factor-α (TNF-α), prostaglandin E2 (PGE_2_), and cytokines IL-1β and IL-6. Moreover, flavonoids suppress inflammation by inhibition of enzymes responsible for superoxide anion production as well as phospholipase A2, cyclooxygenase, and lipoxygenase [4,5,6]. They can also act as scavengers of reactive oxygen species and reactive nitrogen species. Similarly, plant pentacyclic triterpenes possess strong anti-inflammatory activity. The mechanism of their action is based on their capacity of blocking nuclear factor-κB (NFκB), reducing NO production, and inhibiting the release of pro-inflammatory cytokines, such as IL-6, IL-8, IL-1β, and TNFα [7,8,9,10]. Moreover, triterpene acids isolated from frankincense were found to have anti-inflammatory activity based on the suppression of prostaglandin E2 synthase (mPGES-1) [11].

The *Rhododendron* genus belonging to the *Ericaceae* family comprises over 850 species, distributed mainly in the northern hemisphere. The plants are deciduous or more often evergreen shrubs or trees. Many species of this abundant group are used in traditional medicine. Previous studies have shown that extracts from rhododendrons exert significant pharmacological effects [12,13,14].

*Rhododendron luteum* Sweet (RL; lat. *Azalea pontica* L. or *Rhododendron flavum* G. Don; common name yellow azalea or honeysuckle azalea) is a member of the *Rhododendron* genus. In Europe, it is native to southern Poland, Austria, Georgia, Slovenia, and Ukraine. The main growing place of *R. luteum* in Poland is located in Kołacznia nature reserve. RL is a 4 m high deciduous shrub. The plant blooms in May. Its yellow hermaphroditic flowers are pollinated by bees and bumblebees. The leaves are oblanceolate, covered by flat and stiff hairs [15].

In vitro assays performed using human cancer cell lines (HepG2 and WiDr) have revealed that *R. luteum* possess selective anticancer potential [16]. The essential oil isolated from this plant displays antibacterial activity against *S. aureus*, *E. faecalis*, and *S. marcescens* [17]. *R. luteum* has also been reported to have antifungal potential due to the high linalool content [18,19].

As shown by ethnobotanical data, the *Rhododendron* genus may be a rich source of pharmaceutical active compounds. *R. luteum* has been used in Turkish traditional medicine against inflammation and rheumatic pain; supplied externally, it has helped in treatment of fungal infections [14]. Despite such a promising potential use, there is quite limited information about active compounds contained in RL.

Therefore, the aim of our study was to analyze the biological potential and chemical composition of several extracts prepared from *R. luteum* leaves with the accelerated solvent extraction (ASE) technique using different solvents and extraction temperatures. We wanted to verify whether in vitro assays would confirm the ethnobotanical data and to indicate the best conditions of extraction of active metabolites. We focused on mechanisms associated with the inflammatory process and oxidative stress, e.g., influencing the activity of lipoxygenase (one of the pro-inflammatory enzymes), radical scavenging activity, and ion chelating capacity. Moreover, the content of the major groups of active secondary metabolites (including polyphenols and triterpenes) was measured. In addition, detailed qualitative and quantitative analysis of polyphenolic acids, flavonoid aglycones, and flavonoid glycosides was performed using liquid chromatography/electrospray ionization triple quadrupole mass spectrometry (LC-ESI-MS/MS).

## 2. Materials and Methods

### 2.1. Chemicals and Apparatus

Methanol, FeCl_2_, glacial acetic acid, and sodium carbonate anhydrous were purchased from Avantor Performance Materials Poland (Gliwice, Poland). Trolox, gallic acid, 2,2′-azino-bis-3(ethylbenzthiazoline-6-sulphonic acid) (ABTS^•+^) and 2,2-diphenyl-1-picrylhydrazyl (DPPH^•^), Folin-Ciocalteu reagent, 2,2′-azobis (2-methylpropionamide) dihydrochloride (AAPH), ferrozine, vanillin, oleanolic acid, aluminum chloride, caffeic, 5-*O*-caffeoylqunic, *m*-coumaric, *o*-coumaric, *p*-coumaric, gallic, ferulic, isoferulic, protocatechuic, 3-hydroxybenzoic, 4-hydroxybenzoic, vanillic, syringic, salicylic, rosmarinic acid, rutin, hyperoside, isoquercetin, vitexin, isovitexin, quercitrin, 3-*O*-methylquercetin, kaempferol, luteolin, taxifolin, and LC-MS grade acetonitrile were obtained from Sigma-Aldrich Chemical Co. (St. Louis, MO, USA). Perchloric acid was from Chempur (Piekary Śląskie, Poland). Catechin, luteolin-7-*O*-glucoside, luteolin 3′,7′-diglucoside, naringenin 7-*O*-glucoside, gentisic, sinapic acid, eriodictyol, and myricetin were from ChromaDex (Irvine, CA, USA). Astragalin, apigenin, naringenin, kaempferol-3-rutinoside, tiliroside, and fluorescein sodium salt were from Roth (Karlsruhe, Germany) and quercetin was from Fluka (Buchs, Switzerland). LC-MS grade water was prepared using a Millipore Direct-Q3 purification system (Bedford, MA, USA). The Lipoxygenase Inhibitor Screening Assay Kit was obtained from Cayman Chemical (Ann Arbor, MI, USA). Accelerated solvent extractions were conducted in an ASE 150 extractor from Dionex Corporation (Sunnyvale, CA, USA), evaporation of extracts was conducted using a Heidolph Basis Hei-VAP Value evaporator (Schwabach, Germany), and lyophilization was done in the Free Zone 1 apparatus (Labcono, Kansas City, KS, USA). Colorimetric and fluorescence measurements were performed on 96-well transparent and black microplates (both from Nunclon, Nunc; Roskilde, Denmark) respectively, using an microplate reader Infinite Pro 200F from Tecan Group Ltd. (Männedorf, Switzerland).

### 2.2. Plant Material

Leaves of *Rhododendron luteum* Sweet (RL) were collected in Kołacznia nature reserve (Nowa Sarzyna, Poland) in June 2016. Sampling permission was granted by RDOŚ (Regional Director for Environmental Protection) in Rzeszów. The plant material was dried at ambient temperature. A voucher specimen was deposited at the Chair and Department of Pharmaceutical Botany, Medical University of Lublin, Poland (voucher No. RL-01/16). The leaves were powdered and sifted through a sieve (aperture dimension: ø1.6 mm and ø0.28 mm). Grains with a size between ø1.6 and ø0.28 mm were selected for further studies.

### 2.3. Extraction

Nine different types of extracts were obtained from RL using the accelerated solvent extraction system (ASE). Extractions were performed at three temperatures (40, 80, 140 °C) using three solvents: methanol, 80% methanol, and water. The extraction procedure was as follows: 2 g of pulverized plant material mixed 1:1 with diatomaceous earth was loaded into an extraction cell, the cell was filled with solvent up to a pressure of approximately 100 bars, extraction was performed for 15 min, the cell was rinsed with 60% cell volume using an extraction solvent, the solvent was purged from the cell with nitrogen, and depressurization took place. The procedure was conducted three times for each sample. Eluates were combined and filled up to the volume of 50 mL to unify the sample volume and concentration. All extracts were prepared in triplicate. A portion (2 mL) of each freshly prepared extract was taken for analyses of the chemical composition and biological activity. The remaining part of the extract was evaporated, lyophilized, weighed, and kept at −18 °C. Each sample was marked with the solvent and temperature symbol (e.g., RL140C-80M—a sample obtained by extraction with 80% methanol at 140 °C). Then, each sample was carefully diluted in accordance with the specific method requirements just before analysis.

### 2.4. Total Phenolic Content

Total phenolic content was determined with the Folin-Ciocalteu reagent using a method adapted to the microscale [20]. Briefly, 20 μL of Folin-Ciocalteu reagent diluted with water 1:4 (*v/v*) and 160 μL of sodium carbonate (75 g/L) were added to each well containing 20 μL of the sample or standard solution. The mixtures were incubated for 20 min. Absorbance was read at 760 nm using a microplate reader with methanol instead of extract as a blank. Triplicate measurements were made for each sample and averaged. The results were determined as mg of gallic acid (GA) per g of dry extract using the calibration curve prepared for GA solutions at concentrations ranging from 25 to 200 µg/mL.

### 2.5. Total Flavonoid Content

Total flavonoid content was assayed with a modified Lamaison and Carnart method [20,21]. Briefly, 20 μL of the extract was mixed with 160 μL of methanol and 20 μL of a 2% (*v*/*v*) AlCl_3_ solution. The mixtures were shaken and incubated at 28 °C for 30 min. Absorbance was measured at 430 nm with a solution containing methanol instead of the sample as a blank. All measurements were performed in triplicate for each sample. The results were expressed as mg of quercetin per 1 g of dry extract using a standard curve prepared in the same conditions for a series of dilutions (2–20 µg/mL) of the quercetin solution.

### 2.6. Total Triterpene Content

Total triterpene content was estimated using a modified Zhang method [22]. Aliquots (25 μL) of the extract were evaporated by heating in a water bath at 50 °C for 25 min. After evaporation, 100 μL of methanol, 150 μL of a 5% (*v*/*v*) vanillin solution in acetic acid, and 300 μL of 70% (*v*/*v*) perchloric acid were added to the dry extract. The mixture was heated in a 65 °C water bath for 30 min and cooled on ice for 12 min. Then, 2 mL of glacial acetic acid was added to each sample. The samples were shaken and allowed to stand at room temperature for 10 min. Part (300 μL) of each reaction mixture was transferred into a microplate well and the absorbance was measured at 550 nm. Methanol was used instead of the extract in the negative control. Measurements were taken in triplicate. The results were expressed as mg of oleanolic acid per 1 g of dry extract calculated from a standard curve prepared in the same conditions using a series of dilutions (250–2500 µg/mL) of standard solutions.

### 2.7. LC-ESI-MS/MS Analysis of Phenolic Compounds

Qualitative and quantitative analysis of polyphenolic compounds in the samples was performed using high-performance liquid chromatography coupled with triple quadrupole tandem mass spectrometry (LC-MS/MS). The previously used methods were combined and modified to design one method adapted for simultaneous analysis of phenolic acids, flavonoid aglycones, and flavonoid glycosides [23,24,25]. Separations were carried out using an Agilent 1200 Series LC system (Agilent Technologies, USA) and a 4.6 × 150 mm Agilent Eclipse XDB-C18 column (5 µm; Agilent Technologies, USA). The mobile phase consisted of water containing 0.1% HCOOH (solvent A) and acetonitrile containing 0.1% HCOOH (solvent B). The flow rate was 400 µL min^−1^, and the mobile phase gradient was programmed as follows: 0–1.5 min–13% B; 2–4.5 min–20% B; 5–8 min–25%; 9–11 min 33% B; 13–16 min 60% B; 18–21 min 80% B; 23–28 min 13% B. The injection volume and column temperature were 3 µL and 25 °C, respectively. The LC system was connected to a 3200 QTRAP Mass spectrometer (Sciex, Redwood City, CA, USA) equipped with an electrospray ionization source (ESI) and working in the multiple reaction monitoring (MRM) scan mode. Nitrogen was used as curtain and collision gas. ESI worked in the negative ion mode in the following conditions: capillary temperature 500 °C, curtain gas at 30 psi (pound-force per square inch), nebulizer gas at 55 psi, and negative ionization mode source voltage −4500 V. For each analyte, the optimum parameters of the Multiple Reaction Mode (MRM) were determined in the infusion mode. The optimized instrument settings for product ions of each compound are shown in Table S1 (in the Supplementary Material). Analyst 1.5 software (Sciex, Redwood City, CA, USA) was used for data acquisition and processing. The analytes were identified by comparing retention times and MRM transitions with the parameters from corresponding standards tested in the same conditions. The compounds were quantified on the basis of peak areas of the most intense MRM transitions using the results from calibration curves generated for the corresponding standards.

The LOD (limit of detection) and LOQ (limit of quantification) values were established at a signal-to-noise ratio of 5:1 and 10:1 respectively, based on the results obtained for independent replicates of standard solutions (Table S2 in the Supplementary Material). The samples were filtered through a hydrophilic polytetrafluoroethylene (PTFE) 0.20 μm membrane (Merck, Darmstadt, Germany) syringe filter prior to LC injection.

### 2.8. Determination of Antiradical Potential with DPPH^•^ Assay

The ability to scavenge the 2,2-diphenyl-1-picrylhydrazyl radical (DPPH^•^) was assayed according to a method described previously [26]. Briefly, 180 μL of a DPPH^•^ solution (0.152 mM) was mixed with 20 μL of the extract diluted to different concentrations on 96-well microplates. The samples were shaken and incubated at 28 °C for 30 min. Measurements were taken at 517 nm by a microplate reader.

The antiradical activity of fresh extracts was calculated according to the following equation:

% Reduction = [(AB − AA)/AB] × 100

where AB is the absorption of the blank sample (DPPH^•^ solution and solvent instead of the sample), and AA is the absorption of the analyzed sample with DPPH^•^ reagent.

Using at least seven dilutions of the samples, a dose-response curve was prepared for each extract to determine its EC_50_ value (half maximal effective concentration; concentration of extract necessary to decrease the absorbance of DPPH by 50%). The results were calculated and expressed as standard equivalents using Trolox (TE) based on its EC_50_ values obtained using the same conditions and procedure. All experiments were performed in triplicate.

### 2.9. Determination of Antiradical Capacity with ABTS^•+^ Assay

The ABTS^•+^ assay was performed according to a method described previously [26]. The ABTS stock solution was prepared by mixing 4 mL of 9.33 mM aqueous ABTS and 1 mL of 36.6 mM potassium persulfate (K_2_S_2_O_8_) in an amber glass bottle. The mixture was incubated in the dark for 16 h at room temperature. The ABTS^•+^ working solution was prepared daily by mixing 0.5 mL of the stock solution with 19.5 mL of methanol (final concentration of ABTS^•+^ 0.096 mg/mL). The sample (20 µL) was mixed with 180 µL of the ABTS^•+^ working solution. The mixtures were shaken and incubated at 28 °C. After 6 min of incubation, absorbance was measured at 734 nm. The scavenging activity of the extracts was determined using the following formula:

% Reduction = [(AB − AA)/AB] × 100

where AB is the absorption of the control sample (ABTS^•+^ solution and solvent instead of the sample), and AA is the absorption of the sample with ABTS^•+^ reagent.

Seven dilutions of each extract were examined to plot a dose-response curve and determine the EC_50_ value. The results were expressed as standard equivalents using Trolox (TE; Trolox equivalent) based on its EC_50_ value obtained in the same conditions. Triplicate measurements were made for each sample and averaged.

### 2.10. Metal Chelating Activity

Metal chelating power was determined with the modified method described previously [27]. Samples (200 µL) were mixed with 50 µL of a FeCl_2_ solution (0.4 mM) and shaken for 1 min. Then, 100 µL of a ferrozine solution (1 mM) was added, and the mixture was shaken and left standing at room temperature for 10 min. Absorbance was then measured at 562 nm. The blank contained water instead of the sample. Ethylenediaminetetraacetic acid (EDTA) disodium salt (Na_2_EDTA) was used as a positive control.

The chelating power was calculated using the formula:

% chelating power = [(1 − As/Ac)] × 100

where As—absorbance of the mixture with the sample and Ac—absorbance of the blank control.

The results were expressed as Na_2_EDTA equivalents (mg of Na_2_EDTA equivalent/g of dry extract).

### 2.11. Oxygen Radical Absorbance Capacity (ORAC) Assay

The analysis was performed according to slightly modified method of Dienaitė et al. [28]. A phosphate buffer (75 mM, pH 7.4) was used for all sample dilutions and reagent preparations. The working fluorescein solution (10 nM) was freshly prepared by dilution of its stock solution (100 µM) before each analysis. Aliquots of fluorescein solution (150 µL) and sample or standard solution (25 µL) were added to wells of a 96-well black microplate. The mixtures were preincubated for 20 min at 37 °C. The reaction was initiated by adding 25 µL of 240 mM AAPH solution and the fluorescence was monitored every 90 s (for 120 min) at an excitation wavelength of 485 nm and an emission wavelength of 515 nm. The buffer was used as a blank. The sample activity was expressed as mg of Trolox/g dry extract and all determinations were carried out in triplicate.

### 2.12. Lipoxygenase Inhibitor Screening Assay

The anti-inflammatory activity of *Rhododendron luteum* Sweet leaf extracts was determined using the Lipoxygenase Inhibitor Screening Assay Kit by Cayman Chemical according to the manufacturer’s instruction. Briefly, 10 μL of the sample was mixed with 90 μL of a lipoxygenase solution. The samples were tested in three different concentrations (50, 100, and 200 μg of dry extract in the reaction mixture). After 5 min of incubation at room temperature, a solution of arachidonic acid was added and the plate was shaken for 10 min. After this time, 100 μL of chromogen was added, and the mixture was shaken for another 5 min. Absorbance was read at 500 nm. Assay buffer instead of extract was used in the negative control. NDGA (nordihydroguaiaretic acid) was used as a positive control.

### 2.13. Statistical Analysis

All assays were conducted in triplicate. The results were expressed as mean values with the standard deviation of independent measurements. All calculations were done using Microsoft Excel. Statistical analyses were performed using IBM SPSS software. Differences estimated in one-way analysis of variance (ANOVA) with Tukey’s test at *p* < 0.001 (Table 1) and *p* < 0.05 (Table 2, Table 3 and Table 4) were considered statistically significant. Correlation coefficients between the components and activities of the analyzed extracts were determined using Excel.

## 3. Results and Discussion

Since antioxidant and anti-inflammatory activity are attributed to the presence of polyphenols (including flavonoids) [1,4,29] and triterpenes [7,8,9,10], total phenolic (TPC), total flavonoid (TFC), and total triterpene content (TTC) were determined in all the examined extracts.

### 3.1. Chemical Profiles of Different R. luteum Extracts

Based on the results presented in Table 1, it can be concluded that the temperature and solvent type strongly affect the efficiency of extraction of secondary metabolites from *R. luteum* leaves during accelerated solvent extraction. The amount of dry extracts obtained from the same portion of dry plant material varied significantly (0.12–0.47 g/g dry extract). Temperature was a factor with the greatest impact on the extraction efficiency. In the case of all extractants, the lowest amount of dry extract was obtained at the lowest temperature tested (40 °C). The extraction efficiency increased with the increasing temperature. ASE at the highest temperature (140 °C) resulted in double the amount of dry extract. It was previously reported that high temperature can lead to significant improvement in ASE extraction efficiency, by assisting in breaking down analyte–matrix interactions, increasing the solubility of plant components and encouraging the diffusion of the analyte to the solvent [30]. However, the amount of dry extract is not the only indicator of the quality of the extraction process. The quality (composition and activity) of the final extract may be equally or even more important.

It can be noticed that the composition of the different *R. luteum* extracts varied considerably. TPC in the examined *R. luteum* samples ranged from 5.88 to 25.64 mg of gallic acid/g of dry extract. The highest TPC values were reported for the aqueous (W) and hydro-methanolic (80M) extracts. Water seems to be crucial for obtaining high amounts of polyphenolic metabolites from *R. luteum* leaves (Table 1). This probably results from the fact that the majority of polyphenols in plants exist as glycosides and esters [25]. However, the extraction temperature was a strongly influencing factor as well. Its increased value resulted in higher polyphenol content. TPC values are similar to TPC determined in another *Rhododendron* representative—*R. anthopogonoides* (39.26 mg of gallic acid/g of dry extract) [31]. In turn, Turkish *R. luteum* leaf extract (dimethyl sulfoxide extraction) exhibited higher amounts of polyphenolic compounds (173.2 mg of gallic acid/g of sample) [32].

Similarly, recent investigations of aerial parts of *R. luteum* have shown a higher amount of phenolics in water and methanolic extracts [12]. The differences may be related to the different extraction procedures, geographical location, and environmental factors influencing the specimens.

The TFC of the extracts differed greatly (from 1.79 to 7.59 mg of quercetin/g of dry extract; Table 1). Similarly, the highest TFC was obtained for samples extracted at 140 °C. There were no significant differences between samples obtained with different solvents at the same temperatures. Therefore, temperature seems to be the main determinant of the extraction of flavonoids from *R. luteum* leaves. The TFC values are lower than those reported for *R. luteum* flower extracts and samples prepared from *R. luteum* aerial parts [12,16]. The differences may largely result from geographical location and environmental factors, e.g., differences in the intensity of UV radiation [33].

To our knowledge, there is no report about the total triterpene content in any part of *R. luteum* Sweet. However, the presence of these active metabolites was reported for rhododendrons [34,35]. Therefore, the next step of this study was to carry out quantitative determination of total triterpenes (TTC) in all our *R. luteum* samples. The results of this experiment are shown in Table 1. As can be seen, TTC in the examined *R. luteum* samples ranged from 140.17 to 223.04 mg of oleanolic acid/g of dry extract. It is noteworthy that, in contrast to TPC, the extractions with pure and 80% methanol gave the best results. The most prominent TTC values in the group of the hydro-methanolic samples were obtained at 80 and 140 °C. Extraction with the use of pure methanol gave slightly worse results. Interestingly, in the case of the M and 80M samples, temperature significantly influenced the results of the pressurized solvent extraction of triterpenoids. However, the extraction temperature did not significantly affect the triterpene concentrations in the aqueous samples. The content of triterpenes in the *R. luteum* extracts is comparable to their amount in a highly concentrated extract of olive leaf (*Olea europaea* L.). In comparison to this well-known medicinal plant, which contains 32% of triterpenes in dry extract, the RL extracts contained up to 22.30% [36].

High amounts of polyphenols were previously reported in many *Rhododendron* species. Amongst phenolic acids known for their pharmacological activities, chlorogenic, gallic, and caffeic acids were common in extracts made from different rhododendrons [12,37,38]. Moreover, many flavonoids and their glycosides were identified, i.e., kaempferol, catechin, myricetin, hyperoside, isoquercetin, and astragalin [37,39,40,41]. It is known that extraction conditions strongly influence the elution of plant metabolites [42,43]. Moreover, polyphenols in total and individual polyphenolic compounds can strongly affect the activity of samples. Therefore, we decided to quantitatively and qualitatively evaluate the content of potentially important anti-inflammatory and antioxidant components of RL extracts with the use of the LC-ESI-MS/MS-MRM method. The triple quadrupole mass spectrometer working in the multiple reaction monitoring (MRM) mode ensures high sensitivity and specificity; hence, it is an excellent tool for analyses of complex plant samples [25].

**Figure 1:**
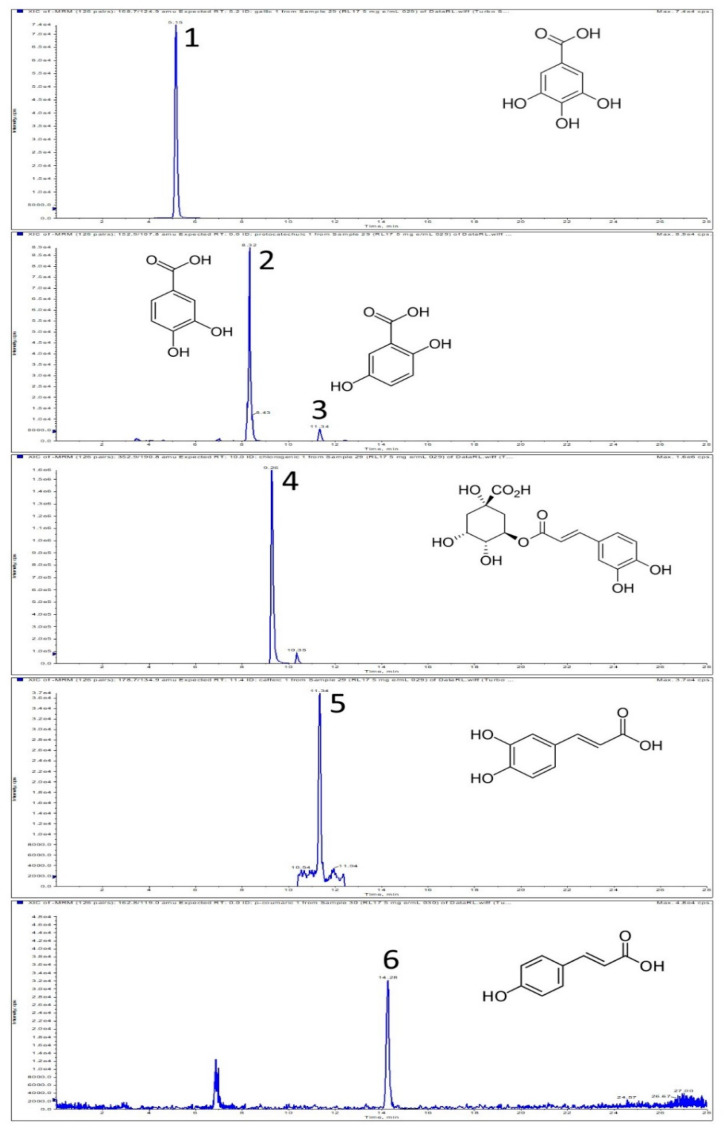
LC-MS (liquid chromatography mass spectrometry) chromatograms obtained in multiple reaction monitoring (MRM) mode of phenolic acids detected in *R. luteum* leaves (sample RL140C-M). Monitored MRM transition is given in the bracket: 1—gallic acid (*m*/*z* 168.7 → 124.9), 2 and 3—protocatechuic and gentisic acids, respectively (*m/z* 152.9 → 107.8). 4—5-*O*-caffeoylquinic acid (*m/z* 352.9 → 190.8), 5—caffeic acid (*m/z* 178.7 → 134.9), 6—*p*-coumaric acid (*m/z* 162.8 → 119). Un-numbered peaks represent uncharacterized constituents.

In this study, twenty-three secondary metabolites representing seven polyphenol subclasses were detected and quantified in the RL samples (Table 2). The presence of five hydroxybenzoic and five hydroxycinnamic acids (gallic, protocatechuic, 5-*O*-caffeoylquinic, 4-hydroxy-benzoic, gentisic, salicylic, caffeic, *p*-coumaric, ferulic, and isoferulic) was shown (Figure 1). Seven flavonoid aglycones: catechin, quercetin, taxifolin, myricetin, eriodictyol, luteolin, and 3-*O*-methyloquercetin (Figure S1 in the Supplementary material) and six flavonoid glycosides: hyperoside, isoquercetin, eriodictyol 7-*O*-glucopyranoside, astragalin, naringenin 7-glucoside, and quercitrin, were identified in the *R. luteum* extracts. There were qualitative and quantitative differences in the phenolic profiles. However, 5-*O*-caffeoylquinic and protocatechuic acids, catechin, and quercetin and its glycosides (hyperoside, isoquercetin, quercitrin) were found to be the dominant compounds in each sample.

It was shown that 5-*O*-caffeoylquinic (one of the chlorogenic acids) was a predominant phenolic acid (4239 to 10,580 μg/g of dry extract). Its content in the extracts prepared with the specified extractants did not differ greatly, indicating that the extraction temperature did not have an enormous impact on its elution from the plant material. In turn, a lower level of this compound was observed in the aqueous samples. Therefore, methanol seems to be necessary for effective extraction of 5-*O*-caffeoylquinic acid. Chlorogenic acid was also identified in other rhododendrons (e.g., *R. cinnabarianum*, *R. virgatum*, and *R. lepidotum*), but its content was reported to be more than three times lower than that found in our study [37]. Another phenolic acid present in a substantial amount in all extracts was protocatechuic acid (485.1–939.2 μg/g), with the highest content in the methanolic-aqueous and aqueous samples. Similarly, the amount of this compound in RL was significantly higher than that reported for other rhododendrons by Prakash et al. [37]. In the cited paper, the phenolic profiles of different rhododendron species were shown to vary greatly [37]. The considerably higher levels of polyphenols found in our samples may be also associated with the use of different extraction methods.

Gallic (86.7–348.0 μg/g) and *p*-coumaric (47.1–247.0 μg/g) acids were identified in all examined extracts. The amount of gallic acid increased with the increasing extraction temperature and was the highest in RL140C-80M. Part of gallic acid could be released from glycosides at high temperatures [25]. In turn, the highest level of *p*-coumaric acid was detected in the methanolic and hydro-methanolic samples prepared at 40 °C.

Gentisic acid was present in relatively small quantities (2.8–10.6 μg/g) in extracts obtained at high temperatures, whereas caffeic acid was the most abundant in the methanolic samples. It was observed that the lower temperatures and the high organic solvent concentration increased the elution of this compound from the RL leaves. Salicylic, 4-hydroxy-benzoic, ferulic, and isoferulic acids were detected in trace amounts (below the quantification limit of the LC-ESI-MS/MS method) in some samples. Vanillic, syringic, sinapic, *m*-coumaric, *o*-coumaric and rosmarinic acids were not detected.

Gallic, caffeic, protocatechuic, and chlorogenic (caffeoylquinic and *p*-coumaroylquinic) acids, as well as derivatives of caffeic, ferulic, *p*-coumaric, vanillic, and gallic acids, were previously identified in different rhododendrons, including *R. luteum* [12,37]. However, to the best of our knowledge, the presence of 4-hydroxy-benzoic, salicylic, gentisic, and isoferulic acids in RL leaves is reported here for the first time.

Flavonoids representing flavonols, flavan-3-ols, flavones, flavanones, and dihydroflavonols were detected in the RL samples, with the dominant levels of catechin (flavan-3-ol) and flavonols (quercetin and its derivatives).

Catechin was found to be the most abundant flavonoid aglycone in all extracts, with values varying from 839 to 3470 μg/g of dry extract. As can be seen in Table 2, the highest quantities of this flavonoid were determined in RL140C-M and RL80C-80M.

Quercetin was the second dominant flavonoid aglycone, and its amount detected in almost all samples was substantially higher than the content in other *Rhododendron* species [37]. The level of quercetin in the RL extracts was largely influenced by the extraction temperature. Samples obtained at 140 °C had a few times higher quantities than the other ones. Interestingly, another aglycone—myricetin—was found only in two samples obtained at 140 °C with methanol and 80% methanol, where it was present in large quantities (1567 and 2150 μg/g). Possibly, the significantly increased quantities of quercetin and myricetin in the 140C samples may result from partial hydrolysis of their glycosides at high temperature. An additional support for this hypothesis is the reduced content of hyperoside, isoquercetin, and quercitrin in the RL140C extracts. Almost all RL samples also contained smaller amounts of two other flavonoid aglycones: taxifolin (dihydroflavonol) and eriodictyol (flavanone). The presence of minimal quantities of 3-*O*-methyloquercetin and luteolin was detected as well.

Amongst the detected flavonoid glycosides, hyperoside was present in the largest amount (2038.5–8600 μg/g of dry extract), followed by isoquercetin and quercitrin. Large quantities of quercetin glycosides were previously detected in RL and other *Rhododendron* representatives, e.g., *R. molle* [12,44]. Rutin and quercetin 3,7-dirhamnoside were not observed in our study. As suggested by Prakash et al. [37], rutin is not typical of all *Rhododendron* species.

Relatively small amounts of kaempferol, apigenin, and their derivatives were previously observed in rhododendron samples [12,37,44,45]. We did not detect free kaempferol and apigenin in RL. However, trace amounts of astragalin (kaempferol 3-*O*-glucoside) were observed. Probably further studies including acid and alkaline hydrolyses may be needed to determine the full flavonoid profile of RL leaves. Similarly, naringenin-7-glucoside and eriodictyol-7-*O*-glucopyranoside were found, but below their quantification limits. Therefore, our observations are in agreement with the previous work indicating the presence of naringenin and eriodictyol derivatives in *R. luteum* leaves [12].

Summing up, the extraction temperature and solvent type influence the polyphenol profile in RL extracts. However, it is impossible to establish one optimal set of extraction conditions for all compounds. Mahomoodally et al. [12] observed the highest amounts of polyphenols in aqueous and methanolic samples. We have found methanolic-water extracts to be the richest sources of polyphenols. As a result of phytochemical analyses, a report presenting the total triterpene content and polyphenolic profile of different RL extracts was elaborated. The presence of many biologically highly active metabolites (e.g., 5-*O*-caffeoylquinic, protocatechuic, gallic, and *p*-coumaric acids, and catechin, quercetin and its derivatives) was revealed or confirmed. Our phytochemical findings can be valuable for the purpose of fingerprint analyses, chemotaxonomic studies, isolation of metabolites, or comparison of RL samples from different geographical regions.

### 3.2. Results of Bioassays

Different organs of rhododendrons have been shown to be promising sources of biologically active metabolites, including antioxidants and anti-inflammatory compounds [12,13,45,46,47,48,49]. However, to the best of our knowledge, all the available studies were carried out for species growing in Turkey, Indonesia, China, India, South Korea, or Taiwan. No investigations of *R. luteum* growing wild in Poland have been performed to date. 

The antioxidant potential of the different RL leaf extracts was tested using four spectrophotometric methods, i.e., ABTS^•+^ radical scavenging assay, DPPH^•^ radical scavenging method, ORAC assay, and determination of metal chelating power.

The results indicate that all extracts exhibited high ability to quench ABTS^•+^ and DPPH^•^ radicals in a concentration-dependent manner (Table 3). The TE (Trolox equivalent antioxidant capacity determined with DPPH^•^ radical) and TE (Trolox equivalent antioxidant capacity determined with ABTS^•+^) values were found to be quite high, which indicates that a large amount of the pure standard has activity equivalent to that of the extract. It was also observed that the antiradical potential varied considerably depending on the solvent and the extraction temperature.

In the case of the DPPH^•^ assay results, the TE values were found to be in the range from 234.18 to 621.90 mg TE/g of dry extract. In general, the best results were obtained using extraction at the higher temperatures of 80 and 140 °C.

The TE values for the pure methanol extracted samples ranged from 275.00 to 537.42 mg/g for extracts prepared at 40 and 140 °C, respectively. Similar values were obtained when 80% methanol was used. In both cases (methanolic and hydro-methanolic extracts), the antiradical potential increased with the increasing extraction temperature. Therefore, 140 °C was found to be the optimal temperature for effective elution of antiradical compounds from the plant material.

Slightly different results were observed for the aqueous samples. The lowest amount of antioxidants were eluted at 40 °C. The highest antiradical activity was observed for the aqueous sample extracted at 80 °C (TE = 621.90 mg/g). The highest temperature tested did not yield better results. On the contrary, a significant decrease was observed in comparison with RL80C-W.

TE values found in a previous study of methanol and water extracts from the aerial parts of *R. luteum* [12] are similar to those reported in our study (381.07–480.07 mg/g). The authors also observed lower activity for water extracts prepared with boiling water. This may suggest that some active compounds from the aqueous extract are decomposed during extraction at 140 °C.

It is worth emphasizing that *R. luteum* leaf extracts have similar antioxidant activity as some well-known antioxidant plant products [50]. TE of RL80C-W (621.90 ± 1.67 mg TE/g) is equivalent to 2484.7 µmol Trolox/g.

In the case of results of the ABTS^•+^ assay, the TE values for the extracts ranged from 88.79 to 349.41 mg/g (Table 3). The highest ABTS^•+^ antiradical activities were exhibited by the aqueous extracts. In this group, the TE values ranged from 285.71 to 349.41 mg TE/g, with the strongest scavenging effect determined for the extract obtained at 80 °C. This result corresponds with the results of the DPPH assay, where RL80C-W was also found to be the most active extract.

The TE values for the hydro-methanolic samples were in the range from 108.87 to 312.83 mg/g and the best result was again obtained for the sample extracted at 80 °C. The corresponding methanolic extracts had lower antiradical capacity. However, as can be seen in Table 3, the increasing temperature in this group of samples substantially increased the efficiency of extraction of antioxidant components.

Based on the presented values, it can be concluded that the optimal temperature for obtaining the most active antiradical extracts from *R. luteum* leaves ranges between 80 and 140 °C. The results obtained in the DPPH^•^ test do not fully correlate with those obtained in the ABTS^•+^ assay. However, in both cases, water (RL80C-W) was found to be the most promising extractant.

The antioxidant activity of rhododendron leaves, e.g., in *R. oldhamii* Maxim. [47] and *R. arboreum* Sm. [46], was reported in some previous studies. Moreover, significant antiradical activity of water-soluble components (procyanidins) was found [48]. Compounds isolated from *R. formosanum* had high antiradical potential, similar to that of RL80C-W (TE = 621.90 mg/g) [48]. Extracts from *R. anthopogonoides* aerial parts showed approximately three times lower activity than RL80C-W in the DPPH^•^ assay and more than four times lower activity than RL80C-80M in the ABTS^•+^ assay [31]. Therefore, the leaves of the Polish RL have been found to be a rich source of free radical scavenging compounds.

Antioxidant activity was also determined using the ORAC assay. ORAC measures the peroxyl radical scavenging capacity by monitoring of the degree of inhibition of peroxyl radical-induced oxidation. The reaction is monitored kinetically. Therefore, the protective effect of antioxidants is determined more precisely, and an underestimation can be prevented [28]. The strongest antioxidant effect was determined for the methanolic and hydro-methanolic extracts prepared at 80 and 140 °C (940.99–1011.59 mg TE/g dry extract). Samples obtained at 40 °C and water extracts showed noticeably lower activity.

Since the antioxidant potential is also related to ion chelating capacity, we decided to determine the chelating power of nine different RL extracts. Na_2_EDTA equivalents were calculated based on the Na_2_EDTA standard curve. It was impossible to determine the EC_50_ values, because the dark color of the concentrated samples (concentration > 5 mg/g of dry extract) disturbed the spectrophotometric measurement. The examined extracts were characterized by quite low or moderate ion chelating ability. It was found that water extract of *S. perfoliata* had approximately ten times higher activity than the RL aqueous samples [51]. Similarly, decoctions and infusions made from different *Crataegus* species were ten times more active than our aqueous extracts [52]. However, in both cited papers, methanolic extracts exhibited similar ion chelating activity to that observed for the RL methanolic samples.

In Turkish folk medicine, *Rhododendron luteum* Sweet aerial parts can be used against inflammation-related diseases like rheumatism or lung disorders [14]. Moreover, examination of some other *Rhododendron* species proved their anti-inflammatory activity in in vivo and in vitro assays. Extracts obtained from rhododendrons reduced NO release in inflamed tissues and inhibited COX-2 expression [49,53,54]. In addition, the extracts effectively alleviated edema in murine tissues and reduced symptoms of psoriasis-like skin inflammation [55,56]. However, the anti-inflammatory activity of RL leaves has not been investigated to date.

To test anti-inflammatory properties of *Rhododendron luteum* Sweet leaf, an experiment with one of the enzymes involved in development of inflammation was conducted. Therefore, the direct ability of the extracts to inhibit lipoxygenase (LOX) activity was studied.

In the preliminary experiments, the RL extracts were investigated at three concentrations (50, 100, and 200 μg of dry extract/mL of reaction mixture). However, interferences were observed at the concentration of 100 and 200 μg/mL; hence, they will not be discussed. The samples at the concentration of 50 μg/mL of the reaction mixture yielded valuable and meaningful results.

As shown in Table 4, significant differences in the LOX inhibition were observed. Water and 80% methanol were found to be the best solvents for extracting active LOX-inhibitors from R. luteum leaves (58.93–90.13% of enzyme inhibition). The methanolic samples were characterized by worse results (23.07–51.17%). In the case of the M extracts, the higher temperature contributed to greater efficiency of the anti-LOX extracts.

As shown by our findings, the aqueous RL samples (67.50–90.13% of LOX inhibition) had higher anti-LOX activity than *R. arboreum* aqueous extracts (48.35% of LOX-inhibition) [57]. The enzyme-inhibition grade of our most active sample—RL80C-W—is comparable to that of the most active ethyl-acetate extract of *R. arboreum* [57]. The water and hydro-methanolic extracts prepared at 80 °C inhibited LOX most effectively. The increase in the extraction temperature to 140 °C reduced the activity. This effect may be related to the degradation of active compounds at a higher temperature. Ribeiro et al. [58] reported that flavonoids demonstrated anti-LOX activity in an in vitro test. The high anti-inflammatory activity of RL80C-W and RL80C-80M may be to some extent related to the high flavonoid content in the aqueous and hydro-methanolic samples. The result found for RL80C-W (90.13%) is extremely promising, since it is comparable to the one observed for pure polyphenolic compounds occurring naturally in *Melicope pteleifolia* (Champ. ex Benth.) T.G. Hartley (94.3%) [59] and for the standard compound nordihydroguaiaretic acid (NDGA).

Our paper describes the biological activity of different RL extracts. It suggests some favorable conditions for obtaining the most effective samples. Therefore, it provides important information for further research and indicates the most promising extracts for in vivo studies.

### 3.3. Correlation between the Antioxidant and Anti-LOX Activities and the Content of Secondary Metabolites

Plant extracts are complex mixtures with many groups and subgroups of potentially active components. It can be observed that the final biological potential of a plant sample results from the activity of individual ingredients or may be related to the synergistic or antagonistic effects of many secondary metabolites [60]. Therefore, we decided to investigate the relationship between the biological activity and chemical composition of the extracts. Pearson’s correlation coefficients were calculated between the amounts of the determined compounds and the DPPH^•^, ABTS^•+^, ORAC, chelating power, and anti-LOX results (Table 5).

The antiradical activity (TE values determined in DPPH^•^ assay) was found to be significantly related to the sum of polyphenolic compounds and the sum of flavonoids (with particular emphasis on flavonoid aglycones; 0.619). It was observed that the extracts with the highest levels of gallic acid, catechin, and quercetin were the most powerful radical scavengers. This is in agreement with the results of some previous studies [20,42]. TTC had a smaller, but also noticeable impact on the TE values. Lower correlation coefficients were observed for the TE values obtained with the ABTS^•+^ assay, which is probably related to the increased sensitivity of ABTS to lipophilic antioxidants, e.g., carotenoids [61]. However, a significant correlation with the catechin level was noticed (0.663). Our observations are in agreement with previous papers, where many polyphenolic metabolites were described to be highly effective radical scavengers [62].

The ORAC results were found to be significantly correlated with the total triterpene content (0.755). Moreover, the correlation was observed between the content of particular flavonoids, total flavonoid content, and gallic acid level to ORAC value. Differences in correlation coefficients obtained for antiradical assays and ORAC are most likely related to different mechanisms of antioxidant activity measured in these tests. Probably, triterpenes are effective inhibitors of peroxyl radical-induced oxidation rather than effective radical scavengers.

Chelating power was found to be correlated with the amount of the particular subgroups (phenolic acids and flavonoid aglycones) and individual polyphenolic compounds. 5-*O*-caffeoylquinic acid and *p*-coumaric acid represented phenolic acids with the greatest effect, while quercitrin, taxifolin, eriodictyol, hyperoside, and isoquercetin were found to be the most relevant flavonoids for metal chelating ability (0.663–0.776). This observation supports the findings of Hider et al. [63]. An impact of the triterpene content was noticeable as well. 

In the case of the anti-LOX potential, the total phenolic and flavonoid contents were observed to exert a considerable impact (0.379 and 0.482 for TFC and TPC, respectively). A particularly evident correlation with the catechin concentration was noticed (0.624), which is in accordance with results of other researchers [4,5,58]. They observed that the catechol group in B-ring (like in catechin) is the vital feature for a potent anti-LOX activity. Interestingly, TTC was not found to affect the activity of this anti-inflammatory enzyme.

## 4. Conclusions

Our research is the first report of the high anti-LOX activity of *Rhododendron luteum* Sweet leaf extracts and indicates conducive conditions for effective extraction of anti-inflammatory compounds from this plant material. The high antioxidant and anti-inflammatory activity are significantly induced by the RL polyphenolic compounds. Triterpenes constitute a great part of secondary metabolites and contribute to the antioxidant and chelating power. However, their role may be underestimated, and further studies are needed to fully evaluate their beneficial potential and the RL triterpene profile. The use of 80% MeOH or water as eluents and elevated temperature yielded the most active radical scavenging and anti-LOX extracts. The LC-ESI-MS-based phytochemical studies revealed great diversity of the polyphenols within several polyphenol classes. Twenty-three compounds were identified and quantified, including phenolic acids and flavonoids that had not been previously reported in RL. It was shown that 5-*O*-caffeoylquinic acid, protocatechuic acid, catechin, and quercetin and its glycosides (hyperoside, isoquercetin, quercitrin), as well as pentacyclic triterpenes, were the dominant secondary metabolites of *R. luteum* leaves.

These findings demonstrate the potential of *R. luteum* extracts as plant material for formulation of products used for prevention or treatment of pathological conditions related to oxidative stress or inflammatory processes. However, further research including toxicity and processing studies are needed to fully evaluate the safety of their use.

## Figures and Tables

**Table 1:** Extraction efficiencies (EX), TPC—total phenolic content, TFC—total flavonoid content, and TTC—total triterpene content in different *R. luteum* extracts. Mean values of three replicate assays with standard deviation. Abbreviations: d.e.—dry extract; d.w.—dry weight; 40C, 80C, and 140C—symbols indicating the extraction temperature; M—methanolic extract; 80M—hydro-methanolic extract (obtained with 80% methanol); W—water extract; e.g., RL140C-80M refers to the extract obtained by extraction with 80% methanol at 140 °C.

Sample	EX (g d.e./g d.w.)	TPC (mg of Gallic acid/g d.e.)	TFC (mg of Quercetin/g d.e.)	TTC (mg of Oleanolic acid/g d.e.)
RL40C-M	0.12 ± 0.01 ^a^	5.88 ± 0.15 ^a^	1.79 ± 0.07 ^a^	140.17 ± 6.73 ^a^
RL80C-M	0.19 ± 0.09 ^b^	10.96 ± 0.14 ^b^	3.54 ± 0.14 ^b^	186.79 ± 0.41 ^b^
RL140C-M	0.29 ± 0.04 ^c^	16.11 ± 0.04 ^c^	6.17 ± 0.48 ^c^	213.96 ± 13.73 ^c^
RL40C-80M	0.16 ± 0.01 ^b^	7.67 ± 0.16 ^a^	2.15 ± 0.16 ^b^	158.33 ± 12.37 ^a^
RL80C-80M	0.26 ± 0.00 ^c^	12.49 ± 0.53 ^b^	4.45 ± 0.16 ^c^	220.38 ± 0.29 ^c^
RL140C-80M	0.40 ± 0.00 ^d^	23.18 ± 0.22 ^d^	6.66 ± 0.21 ^c^	220.38 ± 0.29 ^c^
RL40C-W	0.18 ± 0.02 ^b^	9.66 ± 0.24 ^b^	2.16 ± 0.07 ^b^	152.67 ± 5.66 ^a^
RL80C-W	0.29 ± 0.06 ^c^	15.47 ± 0.20 ^c^	4.13 ± 0.13 ^c^	157.13 ± 0.18 ^a^
RL140C-W	0.47 ± 0.05 ^d^	25.64 ± 0.74 ^d^	7.59 ± 0.95 ^d^	163.48 ± 5.54 ^b^

Mean values of three replicate assays with standard deviation, evaluated by one-way analysis of variance (ANOVA) test with Tukey’s post-hoc test. Subscript letters (a–d) in columns indicate significant differences at *p* < 0.001.

**Table 2:** Content of phenolic acids (μg/g of dry extract), flavonoid aglycons (μg/g of dry extract), and flavonoid glycosides (μg/g of dry extract) in different *R. luteum* extracts. Abbreviations as in Table 1.

Sample	RL40C-M	RL80C-M	RL140C-M	RL40C-80M	RL80C-80M	RL140C-80M	RL40C-W	RL80C-W	RL140C-W
**Hydroxybenzoic acids**									
Gallic	227.0 ± 4.2 ^b^	172.4 ± 3.1 ^a^	251.0 ± 7.1 ^b^	173.3 ± 2.4 ^a^	177.3 ± 1.0 ^b^	348.0 ± 5.7 ^c^	86.7 ± 0.1 ^a^	227.1 ± 4.20 ^b^	273.0 ± 4.2 ^c^
Protocatechuic	785.0 ± 24.0 ^b^	736.0± 5.7 ^b^	731.0 ± 9.9 ^b^	922.0 ± 14.1 ^c^	652.0 ± 11.3 ^b^	878.0 ± 2.8 ^c^	485.1 ± 4.2 ^a^	855.0 ± 9.9 ^c^	939.2 ± 7.1 ^c^
4-hydroxy-benzoic	BQL	0 ± 0	0 ± 0	0 ± 0	0 ± 0	0 ± 0	0 ± 0	0 ± 0	0 ± 0
Gentisic	0 ± 0	0 ± 0	10.6 ± 0.1 ^c^	0 ± 0	0 ± 0	8.2 ± 0.0 ^c^	0 ± 0	2.8 ± 0.0 ^a^	6.8 ± 0.1 ^b^
Salicylic	BQL	0 ± 0	0 ± 0	BQL	BQL	0 ± 0	0 ± 0	BQL	BQL
**Hydroxycinnamic acids**									
5-*O*-caffeoylquinic	9060.0 ± 56.6 ^c^	8810.0 ± 155.6 ^c^	8590.0 ± 14.1 ^c^	10,580.0 ± 141.4^c^	10,030.0 ± 127.3 ^c^	8080.0 ± 46.6 ^b^	6720.0 ± 56.6 ^b^	6910.0 ± 99.0 ^b^	4239.0 ± 86.3 ^a^
Caffeic	126.1 ± 2.4 ^c^	107.9 ± 0.4 ^c^	19.7 ± 0.2 ^a^	83.7 ± 0.4 ^b^	55.1 ± 0.7 ^a^	BQL	BQL	28.4 ± 0.28 ^a^	BQL
*p*-coumaric	247.0 ± 7.1 ^c^	175.5 ± 3.5 ^b^	91.2 ± 1.7 ^a^	244.0 ± 5.7 ^c^	130.6 ± 2.0 ^b^	116.7 ± 1.0 ^b^	47.1 ± 2.7 ^a^	94.7 ± 3.8 ^a^	105.8 ± 0.3 ^a^
Ferulic	BQL	BQL	0 ± 0	BQL	BQL	BQL	BQL	BQL	BQL
Isoferulic	BQL	0 ± 0	0 ± 0	0 ± 0	0 ± 0	0 ± 0	0 ± 0	BQL	0 ± 0
**Dihydroflavonols**									
Taxifolin	45.6 ± 0.3 ^c^	38.3 ± 0.1 ^b^	34.7 ± 1.3 ^b^	46.3 ± 0.7 ^c^	37.2 ± 0.6 ^b^	30.3 ± 1.0 ^a^	23.8 ± 0.3 ^a^	28.0 ± 0.7 ^a^	BQL
**Flavan** **-** **3** **-** **ols**									
Catechin	934.0 ± 14.1 ^a^	839.0 ± 15.6 ^a^	3460.0 ± 28.3 ^c^	1689.0 ± 55.2 ^a^	3470.0 ± 70.7 ^c^	2050.0 ± 42.4 ^b^	1938.0 ± 36.8 ^b^	2120.0 ± 0.2 ^b^	2520.0 ± 28.3 ^b^
**Flavanones**									
Eriodictyol 7-*O*-glucopyranoside	BQL	BQL	BQL	BQL	BQL	BQL	BQL	BQL	BQL
Naringenin 7-glucoside	BQL	BQL	0 ± 0	BQL	BQL	0 ± 0	BQL	BQL	0 ± 0
Eriodictyol	23.3 ± 0.7 ^c^	24.1 ± 0.7 ^c^	23.3 ± 1.0 ^c^	22.0 ± 0.6 ^c^	19.1 ± 0.5 ^c^	20.0 ± 0.7 ^c^	7.4 ± 0.2 ^a^	15.7 ± 0.2 ^b^	15.2 ± 5.7 ^b^
**Flavonols**									
Hyperoside	5005.0 ± 21.2 ^b^	7750.0 ± 70.7 ^c^	3670.0 ± 14.1 ^a^	7100.0 ± 84.9 ^c^	8600.0 ± 56.6 ^c^	3205.0 ± 21.2 ^a^	2780.0 ± 28.3 ^a^	3080.0 ± 56.6 ^a^	2038.5 ± 6.4 ^a^
Isoquercetin	2420.0 ± 56.6 ^b^	2980.0 ± 27.3 ^c^	1978.0 ± 2.8 ^b^	3809.0 ± 15.6 ^c^	2970.0 ± 42.4 ^c^	1639.0 ± 1.4 ^a^	1241.0 ± 4.2 ^a^	1780.0 ± 2.8 ^a^	1058.0 ± 2.8 ^a^
Astragalin	BQL	BQL	BQL	BQL	BQL	BQL	BQL	BQL	BQL
Quercitrin	1385.0 ± 9.9 ^b^	1512.0 ± 8.5 ^c^	1179.0 ± 7.1 ^b^	1912.0 ± 67.9 ^c^	1889.0 ± 15.6 ^c^	899.0 ± 4.2 ^a^	847.0 ± 7.1 ^a^	943.0 ± 1.4 ^a^	623.0 ± 7.1 ^a^
Myricetin	0 ± 0	0 ± 0	1567.0 ± 12.7 ^a^	0 ± 0	BQL	2150.0 ± 14.1 ^b^	0 ± 0	0 ± 0	BQL
Quercetin	466.0 ± 8.5 ^c^	467.0 ± 7.1 ^c^	2695.0 ± 63.6 ^d^	394.5 ± 9.2 ^b^	301.0 ± 4.2 ^b^	2210.0 ± 42.4 ^d^	BQL	95.6 ± 7.1 ^a^	588.0 ± 5.7 ^c^
3-*O*-methyloquercetin	BQL	BQL	4.7 ± 0.1 ^a^	BQL	BQL	BQL	0 ± 0	BQL	BQL
**Flavones**									
Luteolin	BQL	BQL	BQL	0 ± 0	0 ± 0	BQL	0 ± 0	0 ± 0	0 ± 0

Mean values of three replicate assays with standard deviation, evaluated by one-way ANOVA test with Tukey’s post-hoc test. Subscript letters (a–d) in columns indicate significant differences at *p* < 0.05. BQL—below quantitation limit.

**Table 3:** Antioxidant activity of *Rhododendron luteum* Sweet extracts. The results of 2,2-diphenyl-1-picrylhydrazyl radical scavenging assay (DPPH), antiradical capacity determination with 2,2′-azino-bis-3(ethylbenzthiazoline-6-sulphonic acid) (ABTS) and Oxygen Radical Absorbance Capacity (ORAC) assay are expressed as mg TE (Trolox equivalents)/g of dry extract. The results of chelating power assay are expressed as mg Na_2_EDTA equivalents/g of dry extract. Abbreviations as in Table 1.

Sample	DPPH (mg TE/g)	ABTS (mg TE/g)	ORAC (mg TE/g)	Chelating Power (mg Na_2_EDTA/g)
RL40C-M	275.00 ± 6.57	88.79 ± 2.15	758.65 ± 31.81	6.22 ± 0.29
RL80C-M	287.39 ± 14.77	133.58 ± 7.23	1011.59 ± 50.93	5.28 ± 0.13
RL140C-M	537.42 ± 31.32	232.56 ± 11.21	940.99 ± 25.08	5.84 ± 0.19
RL40C-80M	300.69 ± 0.74	108.87 ± 5.06	495.77 ± 23.27	6.13 ± 0.22
RL80C-80M	414.48 ± 23.43	312.83 ± 4.87	987.42 ± 38.74	7.08 ± 0.13
RL140C-80M	528.29 ± 7.04	217.74 ± 9.11	985.72 ± 33.93	6.44 ± 0.06
RL40C-W	234.18 ± 0.50	285.71 ± 0.86	602.80 ± 27.68	2.76 ± 0.12
RL80C-W	621.90 ± 1.67	349.41 ± 12.73	704.45 ± 26.15	4.13 ± 0.21
RL140C-W	457.37 ± 17.00	312.73 ± 8.98	686.54 ± 19.94	2.78 ± 0.02

Mean values of three replicate assays with standard deviation.

**Table 4:** Inhibition of lipoxygenase (LOX) by *R. luteum* samples tested at the concentrations of 50 μg of dry extract/mL of the reaction mixture. The results are given in % of enzyme inhibition. Abbreviations: NGDA—nordihydroguaiaretic acid; other abbreviations as in Table 1.

Sample	% LOX Inhibition
RL40C-M	23.07 ± 0.65 ^a^
RL80C-M	25.18 ± 0.31 ^a^
RL140C-M	51.17 ± 0.82 ^b^
RL40C-80M	58.93 ± 0.77 ^b^
RL80C-80M	80.66 ± 1.10 ^d^
RL140C-80M	67.45 ± 0.77 ^b^
RL40C-W	67.50 ± 1.09 ^b^
RL80C-W	90.13 ± 0.07 ^d^
RL140C-W	73.49 ± 1.14 ^c^
Standard—NGDA (100 μM/well)	100.00 ± 0.00

Mean values of three replicate assays with standard deviation, evaluated by one-way ANOVA test with Tukey’s post-hoc test. Subscript letters (a–d) in columns indicate significant differences at *p* < 0.05.

**Table 5:** Pearson’s correlation coefficients between the biological activities and concentrations of secondary metabolites in different RL extracts. Abbreviations: total phenolic (TPC), total flavonoid (TFC), total triterpene content (TTC); nd—significant correlation not detected; other abbreviations as in Table 3 and Table 4.

	DPPH	ABTS	ORAC	Chelating Power	% LOX Inhibition
TPC	0.689	0.551	nd	nd	0.482
TFC	0.715	0.492	0.374	nd	0.379
TTC	0.440	nd	0.755	0.541	nd
Sum of phenolic acids	nd	nd	nd	0.889	nd
Sum of flavonoid aglycones	0.619	nd	0.401	0.326	nd
Sum of flavonoid glycosides	nd	nd	nd	0.691	nd
Gallic acid	0.683	nd	0.318	nd	nd
Protocatechuic acid	0.430	nd	nd	nd	nd
5-*O*-caffeoylquinic acid	nd	nd	nd	0.863	nd
*p*-coumaric acid	nd	nd	nd	0.553	nd
Catechin	0.561	0.663	nd	nd	0.624
Quercetin	0.486	nd	0.451	0.367	nd
Hyperoside	nd	nd	0.337	0.663	nd
Isoquercetin	nd	nd	nd	0.686	nd
Quercitrin	nd	nd	0.451	0.733	nd
Taxifolin	nd	nd	nd	0.776	nd
Eriodictyol	nd	nd	0.489	0.767	nd

## Supplementary Materials

The following are available online at https://www.mdpi.com/2076-3921/9/9/822/s1, Table S1: Summary of optimized LC-MS/MS parameters used for the qualitative analysis of phenolic acids and flavonoids in the MRM scan mode, Table S2: Analytical parameters applied in the LC-MS/MS quantitative method, Figure S1: LC-ESI-MS/MS chromatograms (the MRM mode) of flavonoid aglycones found in sample RL140C-M; Monitored MRM transition is given in the bracket: 1—catechin (*m/z* 288.8 → 244.9); 2—taxifolin (*m/z* 302.7 → 124.9); 3—myricetin (*m/z* 316.7 → 136.9); 4—luteolin (*m/z* 284.7 → 132.9); 5—eriodictyol (*m/z* 286.7 → 134.9); 6—quercetin (*m/z* 300.7 → 150.9); 7—3-*O*-methylquercetin (*m/z* 314.7 → 299.8).
