# Supplementary material for: Influence of Accelerated Solvent Extraction Conditions on the LC-ESI-MS/MS Polyphenolic Profile, Triterpenoid Content, and Antioxidant and Anti-lipoxygenase Activity of Rhododendron luteum Sweet Leaves
Source: Antioxidants (Basel). 2020 Sep 3;9(9):822. doi: 10.3390/antiox9090822 (PMC7555744; doi:10.3390/antiox9090822)

SUPPLEMENTARY MATERIAL

Influence of accelerated solvent extraction conditions on the LC-ESI-MS/MS polyphenolic profile, triterpenoid content, and antioxidant and anti-lipoxygenase activity of *Rhododendron luteum* Sweet leaves

Marta Olech*, Lena Łyko and Renata Nowak

Department of Pharmaceutical Botany, Medical University, 1 Chodźki Street, 20-093 Lublin, Poland

* Correspondence: marta.olech@umlub.pl; Tel.: +48 81 448 70 63; Fax : +48 81 448 70 60

Abstract: Evaluation of native plant resources and their efficient use is one of the current trends in phytochemistry. The main aim of the present study was to investigate the biological activities of different *Rhododendron luteum* Sweet leaf extracts obtained with the use of accelerated solvent extraction using different solvents and extraction temperatures. All extracts were subjected to bioactivity assays, which revealed considerable anti-lipoxygenase (23.07–90.13% lipoxygenase inhibition) and antiradical potential. All samples exhibited high 2,2-diphenyl-1-picrylhydrazyl ($DPPH^{\bullet}$) (234.18–621.90 mg Trolox equivalents (TE)/g) and 2,2′-azino-bis-3(ethylbenzthiazoline-6-sulphonic acid) ($ABTS^{\bullet+}$) (88.79–349.41 mg TE/g) scavenging activity, high antioxidant potential in the Oxygen Radical Absorbance Capacity (ORAC) assay (495.77–1011.59 mg TE/g), and moderate ion chelating (Fe^{2+}) capacity. The chemical profile of each sample was determined using liquid chromatography/electrospray ionization triple quadrupole mass spectrometry (LC-ESI-MS/MS) and spectrophotometric procedures. Twenty-three compounds representing seven polyphenol subclasses were detected and quantified, including some phenolic acids and flavonoids that had not been previously reported for this plant material. It was shown that 5-*O*-caffeoylquinic acid, protocatechuic acid, catechin, quercetin and its glycosides (hyperoside, isoquercetin, quercitrin), and pentacyclic triterpenes were the dominant secondary metabolites in *R. luteum* leaves. The antioxidant activity was found to be strongly related to different polyphenol groups and total triterpene content, while the anti-lipoxygenase potential was highly dependent on catechin.

Keywords: honeysuckle azalea; yellow azalea; Ericaceae, anti-inflammatory activity; antioxidants; flavonoids; phenolic acids; triterpenes; LC-MS; ASE

Table S1. Summary of optimized LC-MS/MS parameters used for the qualitative analysis of phenolic acids and flavonoids in the MRM scan mode

Analyte	Retention time [min]	Precursor ion $[M-H]^-$ [m/z]	Product ions [m/z]	Colision energy [eV]
**Phenolic acids - Hydroxybenzoic acid derivatives**				
Gallic acid	5.15	168.7	78.9<br>124.9	-36<br>-14
Protocatechuic acid	8.32	152.9	80.9<br>107.8	-26<br>-38
4-Hydroxybenzoic acid	10.84	136.8	92.9	-18
Gentisic acid	11.32	152.9	80.9<br>107.8	-110<br>-52
Vanillic acid	11.41	166.8	107.9<br>123	-18<br>-12
Syringic acid	11.42	196.9	122.8<br>181.9	-24<br>-12
3-Hydroxybenzoic acid	12.12	136.8	93<br>75	-16<br>-48
Salicylic acid	17.91	136.8	93<br>75	-16<br>-48
**Phenolic acids - Hydroxycinnamic acid derivatives**				
5-*O*-caffeoylquinic acid	9.26	352.9	190.8<br>84.9	-24<br>-60
Caffeic acid	11.34	178.7	88.9<br>134.9	-46<br>-16
4-Hydroxycinnamic acid (*p*-coumaric acid)	14.27	162.7	119<br>93	-14<br>-44
Sinapic acid	14.47	222.8	121<br>148.9	-36<br>-20
Ferulic acid	14.80	192.8	133.9<br>177.9	-16<br>-12
Isoferulic acid	15.22	192.8	133.9<br>177.9	-16<br>-12

3-Hydroxycinnamic acid (*m*-coumaric acid)	15.50	162.7	119<br>91	-14<br>-36
Rosmarinic acid	15.91	358.7	160.8<br>196.8	-20<br>-22
2-Hydroxycinnamic acid (*o*-coumaric acid)	16.80	162.7	119<br>93	-14<br>-46
**Dihydroflavonols**				
Taxifolin (dihydroquercetin)	15.18	302.7	124.9<br>284.8	-26<br>-14
**Flavan-3-ols**				
Catechin	9.67	288.8	244.9<br>109	-16<br>-32
**Flavanones**				
Eriodictyol 7-*O*-rutinoside	11.93	594.8	286.9<br>150.9	-34<br>-46
Eriodictyol 7-*O*-glucopyranoside	13.06	448.8	286.9<br>134.9	-24<br>-48
Naringenin 7-*O*-glucoside	15.12	432.7	270.8<br>118.9	-22<br>-64
Eriodictyol	17.91	286.7	134.9<br>150.9	-32<br>-18
**Flavonols**				
Quercetin 3,7-dirhamnoside	11.35	592.8	445.7<br>298.9	-48<br>-34
Rutin (Quercetin 3-*O*-rutinoside)	11.99	608.7	299.6<br>270.9	-46<br>-60
Hyperoside (Quercetin 3-*O*-galactoside)	12.80	462.7	299.7<br>254.7	-28<br>-42
Isoquercetin (Quercetin 3-*O*-glucoside)	13.00	462.7	299.7<br>270.7	-30<br>-44
Kaempferol 3-*O*-rutinoside	13.31	592.7	284.8<br>226.7	-38<br>-68
Astragalin (Kaempferol 3-*O*-glucoside)	14.66	446.7	226.8<br>254.8	-54<br>-40

Quercitrin (Quercetin 3-*O*-rhamnoside)	14.83	446.7	299.7<br>270.7	-30<br>-40
Myricetin	16.68	316.7	136.9<br>150.9	-32<br>-26
Tiliroside	17.39	592.8	284.8<br>254.7	-38<br>-56
Quercetin	17.94	300.7	150.9<br>178.8	-26<br>-20
3-*O*-Methylquercetin	18.13	314.7	299.8<br>270.8	-18<br>-26
Kaempferol	18.85	284.7	116.8<br>93	-46<br>-52
**Flavones**				
Luteolin 3',7'-diglucoside	11.28	609.1	285<br>447	-50<br>-32
Isovitexin (Apigenin 6-*C*-glucoside)	12.38	430.8	310.9<br>340.9	-28<br>-26
Vitexin (Apigenin 8-*C*-glucoside)	12.40	430.8	310.9<br>340.9	-26<br>-34
Luteolin 7-*O*-glucoside	12.87	446.8	284.8<br>132.9	-30<br>-78
Luteolin	17.85	284.7	132.9<br>150.9	-38<br>-26
Apigenin	18.64	268.8	117<br>106.8	-44<br>-34

Table S2. Analytical parameters applied in the LC-MS/MS quantitative method

Analyte	LOD [ng/mL]	LOQ [ng/mL]	R^2	Linearity range [ng/mL]
**Hydroxybenzoic acids**				
Gallic acid	500	750	0.9989	750-17500
Protocatechuic acid	300	500	0.9987	1900-19000
4-Hydroxybenzoic acid	200	250	0.9993	770-19250
Gentisic acid	100	200	0.9993	2050-19900
Vanillic acid	1000	1830	0.9985	1830-18300
Syringic acid	500	732	0.9991	732-18300
3-Hydroxybenzoic acid	400	700	0.9996	1700-34000
Salicylic acid	600	800	0.9984	1800-18000
**Hydroxycinnamic acids**				
5-O-caffeoylquinic acid	100	200	0.9991	200-18000
Caffeic acid	195	389	0.9992	389-19500
p-coumaric acid	83	200	0.9989	415-13800
Sinapic acid	500	800	0.9992	800-38000
Ferulic acid	1300	1900	0.9987	1900-36000
Isoferulic acid	1000	1600	0.9990	1800-36000
m-coumaric acid	75	190	0.9993	1890-37800
Rosmarinic acid	150	198	0.9986	396-13200
o-coumaric acid	20	40	0.9987	2000-20000
**Dihydroflavonols**				
Taxifolin	20	40	0.9988	40-1000
**Flavan-3-ols**				
Catechin	225	350	0.9987	350-6500
**Flavanones**				
Eriodictyol 7-*O*-rutinoside	70	120	0.9986	800-25000
Eriodictyol 7-*O*-glucopyranoside	100	250	0.9991	1670-50000
Naringenin 7-*O*-glucoside	100	170	0.9987	250-25000

Eriodictyol	40	70	0.9986	70-6600
**Flavonols**				
Quercetin 3,7-dirhamnoside	100	250	0.9985	1670-25000
Rutin	125	300	0.9983	1500-22000
Hyperoside	167	250	0.9983	500-25000
Isoquercetin	167	250	0.9988	2500-50000
Kaempferol 3-*O*-rutinoside	60	120	0.9992	120-60000
Astragalin	100	250	0.9988	1670-25000
Quercitrin	75	125	0.9986	750-20000
Myricetin	3200	6600	0.9992	6600-66000
Tiliroside	100	250	0.9986	1000-25000
Quercetin	75	125	0.9987	150-6600
3-*O*-Methylquercetin	15	30	0.9991	150-10000
Kaempferol	35	70	0.9990	165-3300
**Flavones**				
Luteolin 3’,7’-diglucoside	250	500	0.9988	1000-22500
Isovitexin	100	250	0.9994	1670-50000
Vitexin	100	200	0.9984	2000-50000
Luteolin 7-*O*-glucoside	50	100	0.9980	250-25000
Luteolin	15	30	0.9979	35-1800
Apigenin	20	30	0.9981	90-4500

Figure S1. LC-ESI-MS/MS chromatograms (the MRM mode) of flavonoid aglycones found in sample RL140C-M; Monitored MRM transition is given in the bracket: 1 – catechin (m/z 288.8 → 244.9); 2 – taxifolin (m/z 302.7 → 124.9); 3 – myricetin (m/z 316.7 → 136.9); 4 – luteolin (m/z 284.7 → 132.9); 5 – eriodictyol (m/z 286.7 → 134.9); 6 – quercetin (m/z 300.7 → 150.9); 7 – 3-O-methylquercetin (m/z 314.7 → 299.8).

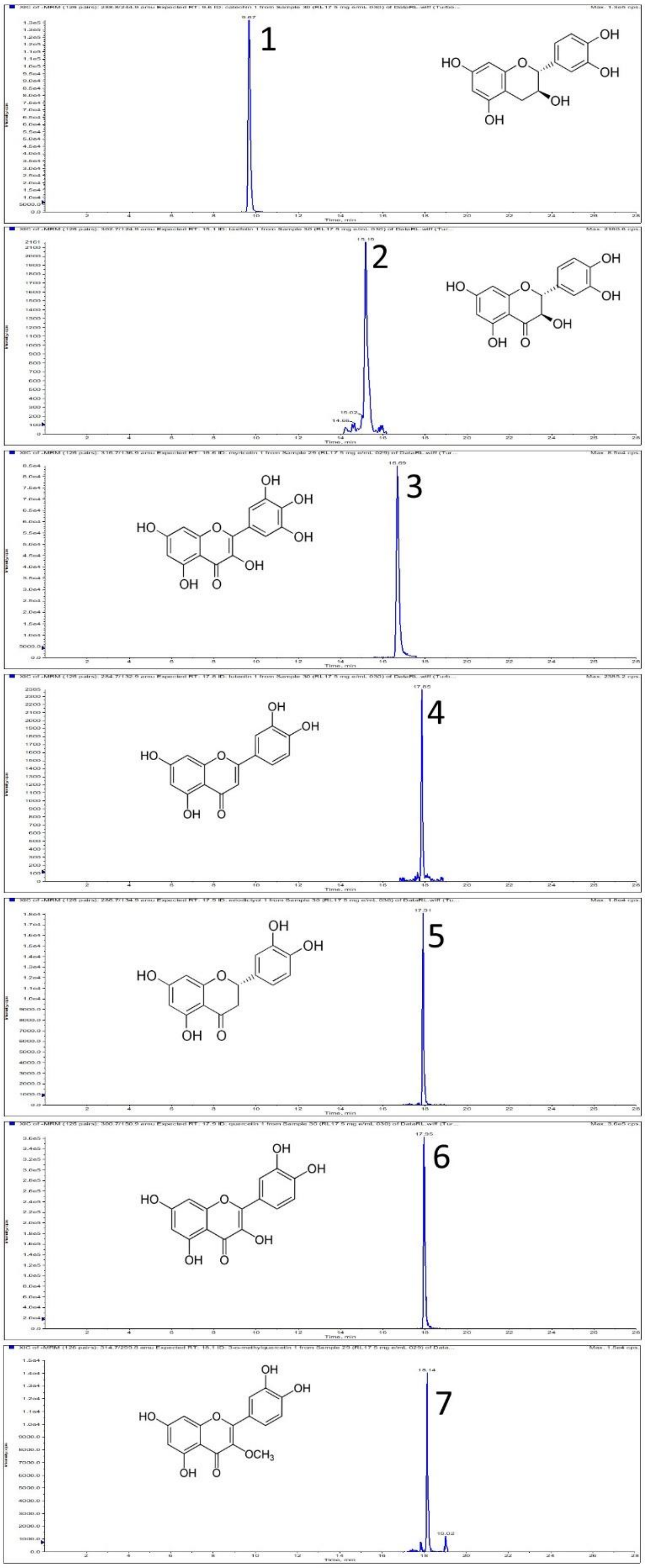

Supplement: Supplementary file 1 [file antioxidants-09-00822-s001.pdf]
